# Patterns of suboptimal antipsychotic use and misuse in Australia: What can routinely collected data tell us?

**DOI:** 10.1111/bcp.15821

**Published:** 2023-07-21

**Authors:** Jonathan Brett, Malcolm B. Gillies, Nicholas A. Buckley, Sallie‐Anne Pearson, Helga Zoega

**Affiliations:** ^1^ Medicines Intelligence in Health, School of Population Health, Faculty of Medicine and Health UNSW Sydney Sydney New South Wales Australia; ^2^ Clinical Therapeutics Department St Vincent's Hospital Sydney NSW Australia; ^3^ New South Wales Poisons Information Centre Westmead Children's Hospital Sydney NSW Australia; ^4^ Clinical Pharmacology and Toxicology Research Group, Biomedical Informatics and Digital Health University of Sydney Sydney Australia; ^5^ Centre of Public Health Sciences, Faculty of Medicine University of Iceland Reykjavík Iceland

**Keywords:** antipsychotic, death, dispensing, latent class analysis, overdose

## Abstract

**Aims:**

There are increasing concerns about harms related to suboptimal antipsychotic use. Here we describe recent population‐based trends in antipsychotic use and harms in Australia and identify population groups exhibiting patterns of use likely to contribute to these harms.

**Methods:**

Using population‐based data from the Australian Pharmaceutical Benefits Scheme (2015‐2020), poisoning calls to the New South Wales (NSW) Poisons Information Centre (2015‐2020) and poisoning deaths in all coronial records (2005‐2018) in Australia, we measured trends in the prevalence of antipsychotic use and related deaths and poisonings. We applied latent class analyses to identify patterns of antipsychotic use that may contribute to harms.

**Results:**

Quetiapine and olanzapine had the highest prevalence of use between 2015 and 2020. Noteworthy trends included increases of 9.1% and 30.8% in quetiapine use and poisonings, while olanzapine use decreased by 4.5% but poisonings increased by 32.7%. Quetiapine and olanzapine poisonings and related deaths had the highest rates of co‐ingestion of opioids, benzodiazepines and pregabalin compared to other antipsychotics. We identified six distinct population groups using antipsychotics: (i) ongoing high‐dose use with sedatives (8%), (ii) ongoing use (42%), (iii) ongoing use with analgesics and sedatives (11%), (iv) long‐term low‐dose use (9%), (v) sporadic use (20%) and (vi) sporadic use with analgesics (10%).

**Conclusion:**

Ongoing potentially suboptimal antipsychotic use and associated harms highlight the need to monitor such patterns of use, for example through prescription monitoring systems.

**What is already known about this subject**

- Antipsychotics are safe and effective when used for on‐label indications such as schizophrenia and bipolar affective disorder.
- Prior studies demonstrate that up to a third of all second‐generation antipsychotic use is off‐label, often for indications with an unclear or unfavourable balance of safety and effectiveness.
- Suboptimal antipsychotic use can increase the risk of harms such as misuse, overdose and death, particularly when used alongside other sedating psychotropics. However, such use and harms has not been previously quantified on a national level in Australia.

**What this study adds**

- Prevalence of use and harms from quetiapine and olanzapine were the greatest of any antipsychotic between 2015 and 2020 and increased over the study period.
- Quetiapine and olanzapine poisonings and related deaths had the highest rates of co‐ingestion of opioids, benzodiazepines and pregabalin compared to other antipsychotics.
- Based on dispensing patterns and demographic information, a substantial proportion of antipsychotic use is likely to be for off‐label indications, some of which may represent misuse, highlighting the need to monitor suboptimal prescribing, for example through prescription monitoring systems.

## 1 INTRODUCTION

Antipsychotics comprise a broad class of medicines effective for managing psychotic disorders, including schizophrenia and bipolar affective disorder.^1^ Evidence supports the use of some antipsychotics to manage generalized anxiety disorder and treatment‐resistant depression.^2^ In recent decades, improved access to mental healthcare has contributed to a worldwide rise in antipsychotic use, driven by increased prescribing of quetiapine, olanzapine and risperidone.^3^ However, there are long‐standing concerns regarding suboptimal antipsychotic use. Harms associated with antipsychotics include weight gain and metabolic syndrome, sudden cardiac death, cognitive decline and falls in older adults, deliberate self‐poisoning and death.^4^, ^5^, ^6^

Concomitant use of antipsychotics with other medicines with sedating effects such as opioids, benzodiazepines and pregabalin may also increase the risk of poisonings and death.^7^, ^8^, ^9^ While the demonstrable effectiveness of antipsychotics in treating schizophrenia and bipolar affective disorder outweighs the potential harms, the risk‐benefit ratio remains unclear or unfavourable for many off‐label conditions. Examples include quetiapine for insomnia, anxiety and behavioural and psychological symptoms of dementia in older adults.^4^, ^10^, ^11^, ^12^, ^13^

Under the umbrella of suboptimal antipsychotic use, misuse, diversion, nonprescribed use and use via nonrecommended routes of administration (eg, intravenous and intranasal) have been well described and are associated with nonfatal and fatal overdose.^14^, ^15^, ^16^ However, most existing reports are derived from populations typically at higher risk of misuse, eg, incarcerated people and people receiving treatment or services related to alcohol and drug abuse.

Latent class analysis (LCA) of dispensing claim‐based indicator data have been applied to opioids, psychostimulants and pregabalin to identify distinct groups of people at risk of suboptimal medicine use.^17^, ^18^, ^19^ This approach allows therapeutic indications and associated patient characteristics to be inferred from dispensing claims in the absence of clinical data. Information derived from LCA can assist in targeting interventions, such as claims data‐based systems to detect and measure suboptimal patterns of medicine use.

Here we describe recent Australian population‐based trends in antipsychotic use, poisonings and associated deaths, identify which antipsychotics are overrepresented in poisonings and deaths, and explore patterns of antipsychotic dispensing indicative of suboptimal use which might be driving harms. To this end, we apply LCA to distinguish groups of people based on their demographics and claims‐based indicators, including time between dispensings, number of prescribers and number of dispensed daily doses.

## 2 METHODS

We leveraged population‐based data from three Australian sources to describe recent trends in antipsychotic use, poisonings and deaths, and used LCA to identify patient characteristics associated with indicators of suboptimal use and misuse.

### 2.1 Data sources

#### 2.1.1 Dispensing claims

All Australian citizens and permanent residents are entitled to subsidized access to certain prescribed medicines via the Pharmaceutical Benefits Scheme (PBS). The PBS 10% sample data set is a random 10% sample of PBS‐eligible Australians and their dispensing claims.^20^ The PBS includes prescription medicines dispensed through community pharmacies, private hospitals and outpatient services, as well as for patients on discharge from public hospitals in all states and territories except New South Wales (NSW) and Australian Capital Territory. The PBS does not capture over‐the‐counter (OTC) medicines or private prescriptions where the patient pays the full cost out of their own pocket. PBS dispensing data contain information on the item dispensed, which is coded by the Anatomical Therapeutic Chemical (ATC) Classification system,^21^ date of dispensing and sex, age and beneficiary status of the recipient, but they lack information on indication and intended dose. General beneficiaries pay a higher co‐payment than their concessional beneficiary counterparts. Concessional beneficiaries are generally people aged ≥65 years, on low incomes, unemployed or with disabilities.^20^

In this study, we used dispensing data for all antipsychotics (ATC N05A) between 1 January 2015 and 31 December 2020 for those 18 years and older.

#### 2.1.2 Poisonings

The NSW Poisons Information Centre (PIC) is Australia's largest PIC, taking approximately 100 000 calls annually.^22^ NSW PIC provides a 24/7 service to healthcare professionals and members of the public, handling calls from NSW, Tasmania and Australian Capital Territory from 6:00 AM to midnight, and providing overnight cover for the whole nation for half the time. The NSW poison centre database records information on caller sex, age group, details of poisonings and advice given to the caller. Drug‐related information is coded at the drug and drug class level (eg, antipsychotics, anxiolytics, hypnotics and sedatives, and opioids). We used all calls related to poisonings with the antipsychotics of interest between 1 January 2015 and 31 December 2020. We included people 15 years and older due to limited capture of actual age. We calculated the number of calls per calendar year stratified by each antipsychotic.

#### 2.1.3 Deaths

The Australian National Coronial Information System (NCIS) database contains a record of all reportable deaths in Australia since July 2000. What constitutes a reportable death differs slightly between Australian states, but all violent or unnatural deaths must be reported. The NCIS carries information including patient demographics and details surrounding deaths contained within a database and attached reports (police, toxicology and autopsy reports, and coronial findings). Drug‐related information is coded and available at the drug level only. We searched the NCIS database for closed cases from 1 January 2015 to 31 December 2018. At the time of data extraction, the national case closure rate dropped from 91.0% for 2018 to 50.7% for 2020^23^ due to ongoing inquests. We extracted all deaths within the NCIS relating to poisonings with antipsychotics and restricted analyses to quality‐assured cases.

### 2.2 Data analysis

#### 2.2.1 Trends in the prevalence of antipsychotic use, poisonings and deaths

We focused on the antipsychotics most commonly dispensed to adult Australians,^24^ ie, quetiapine, olanzapine, risperidone, aripiprazole and clozapine, and grouped all other antipsychotics as “other antipsychotics” (Supporting Information Table S1).

##### Prevalence

We calculated total dispensing and prevalence counts for the entire 6‐year period. For each antipsychotic we calculated the annual prevalence of use (per 1000 population) as the number of people dispensed a PBS‐listed antipsychotic at least once in each year. We used the mid‐year population estimates from the Australian Bureau of Statistics for each year as the denominator.^25^ As our data are based on a 10% sample of the Australian population, we multiplied the prevalence as well as all other population‐based estimates originating from this dataset by 10. We calculated relative changes in prevalence counts as the difference between the 2020 and 2015 rates, divided by the 2015 rate and converted to a percentage.

##### Poisonings

We determined the number of poisonings in 2020 with each antipsychotic in which a drug coded as an opioid, anxiolytic, hypnotic, sedative or pregabalin was also ingested, expressed as a proportion of all calls relating to that antipsychotic.

##### Deaths

For deaths reported in 2018 we determined the number of deaths associated with each antipsychotic in which an opioid, benzodiazepine or pregabalin was also associated as a proportion of all deaths associated with that antipsychotic.

As an exploratory analysis to identify antipsychotics that may be more associated with poisonings or deaths relative to their prevalence of use, we plotted deaths and poisonings against prevalence, using data from the most recent study year available. For poisonings, we plotted national prevalence against calls from the NSW Poisons Centre. We fitted a Poisson regression line to this data using prevalence as an offset, along with 95% prediction intervals.

#### 2.2.2 Latent class analysis

LCA is a statistical modelling technique that takes complex, multivariable data and transforms it into a simpler structure of a few “latent classes” (described elsewhere in this text as “groups”). Each of these groups has its own distinct distribution of indicator values (eg, younger people in one group, older people in another), making it easier to describe different subpopulations. The LCA methods for this study are described in detail in the **Supporting Information**.

We adapted several claims‐based indicators of prescribed medicine use and misuse from previous LCA studies of opioids and pregabalin,^17^, ^18^ and applied these to the PBS 10% dispensing claims. Our indicators were average time between dispensings (only one dispensing, <20 days, ≥20 days), the number of unique antipsychotic prescribers (1‐3 and ≥4) and annual total defined daily doses (DDD) of medicine (<42, 42‐330, >330). In addition, we identified concomitant use of opioids (ATC N02A), benzodiazepines (ATC N05B and N05C) or pregabalin (ATC N03AX16) as a dispensing of one of these medicines within 30 days prior to or following a dispensing of the antipsychotic of interest. Given that off‐label antipsychotic use is frequently at a subtherapeutic dose relative to on‐label indications,^11^ we also identified people for whom only the lowest strength formulation was dispensed. These were only included for individual antipsychotics in which the lowest strength tablet yielded a daily dose below the defined therapeutic range according to the Australian Medications Handbook^1^ (Supporting Information Table S2). We excluded depot formulations due to the lack of applicability of indicators relating to the time between dispensings for these formulations.

We performed LCA using the indicators derived from dispensing claims, grouping the data by calendar year. We chose the optimal number of groups by comparing fit statistics, ie, Akaike's information criterion (AIC), and considering clinical relevance and group prevalence, retaining only models with robust convergence.

After performing the LCA, we characterized the distribution of other covariates of interest within each group to assist in inferring the probable patient types. We assigned a covariate to identify each antipsychotic of interest and those grouped as “other antipsychotic”. We also assigned other covariates that might be associated with group membership, including sex (male, female), age group (18‐39, 40‐64 and 65+ years) and concessional status (general or concessional beneficiary).

For each group, the group‐specific item response probability for each indicator and the aggregate group‐membership probability for each covariate were expressed as percentages. Thus, within each group, the sum of each indicator or covariate percentage was equal to 100%, indicating the distribution of that indicator or covariate within a given group. We tabulated group and covariate distributions for 2020 only as this could vary from year to year, while membership of indicators was invariant and hence tabulated for the entire study period.

Following review by the two clinical pharmacologist authors (J.B. and N.B.), we named the six groups using descriptors with clinical face validity, based on the distribution of indicators and covariates.

#### 2.2.3 Ethics

The NSW Population and Health Services Research Ethics Committee (2013/11/494) granted ethics approval to use PBS data, the Sydney Children's Hospital HREC (2021/ETH00165) to use the NSW Poisons and Justice Health HREC (CF/18/26248) to use NCIS data.

## 3 RESULTS

On a national level, we estimated a total of 23 227 560 antipsychotic dispensings to 947 560 people during 2015‐2020. Of people with an antipsychotic dispensing, 481 980 (50.9%) were female, 735 180 (77.6%) were concessional beneficiaries (people on low incomes, unemployed or having disabilities) and 305 980 (32.3%), 324 650 (34.3%) and 316 930 (33.5%), respectively, were aged 18‐39, 40‐64 and 65+ years at first dispensing.

We observed 18 399 NSW poison centre calls relating to antipsychotics. Of these, 10 745 (58.4%) callers were female and 2495 (13.6%), 15 601 (84.8%) and 302 (1.6%), respectively, callers were aged 15‐19, 20‐74 and 75+ years. Of all calls related to antipsychotics, 11 048 (60.0%) were reported to be intentional self‐poisonings, 10 594 (57.6%) had symptoms relating to the exposure at the time of the call, and 11 737 (63.8%) were either in hospital or referred to hospital as the result of advice given during the call.

Between 2015 and 2018 there were 1908 poisoning deaths associated with antipsychotics: 712 (37.2%) of those deceased were female and 706 (37.0%), 1100 (57.7%) and 102 (5.3%) were aged 18‐39, 40‐64 and 65+ years, respectively. Of all antipsychotic‐related poisoning deaths, 383 (20.1%) were considered intentional, 552 (28.9%) were considered unintentional and intentionality could not be determined in 973 (51.0%) deaths.

### 3.1 Trends in the prevalence of antipsychotic use, poisonings and deaths

**FIGURE 1:**
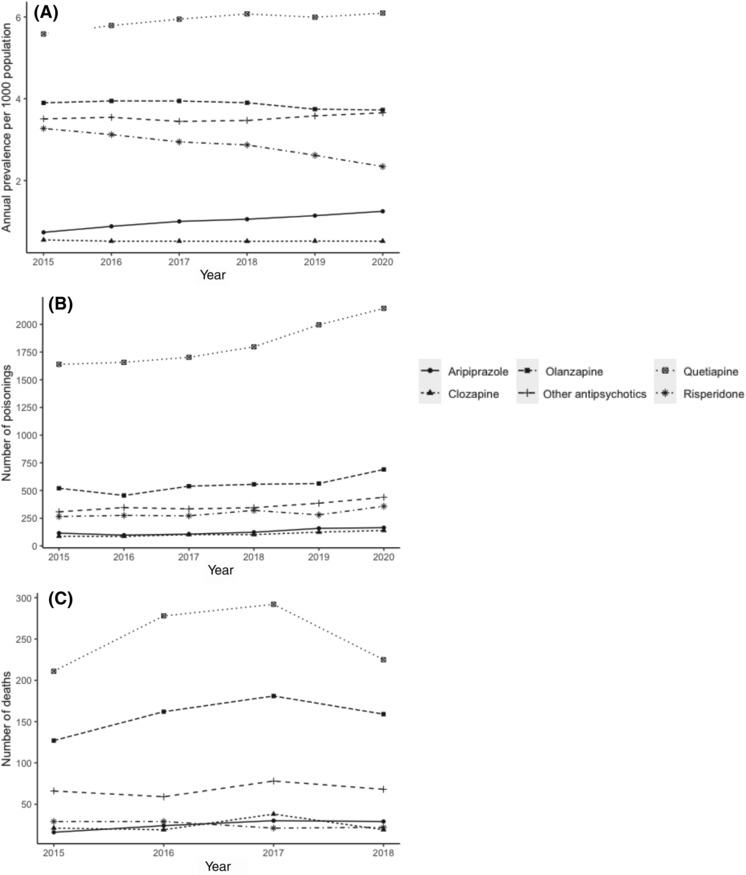
(A) Annual prevalence of dispensing of antipsychotics, expressed per 1000 population 2015‐2020, (B) number of calls to the New South Wales Poisons Information Centre 2015‐20, (C) the number of deaths reported to the coroner within the National Coronial Information System 2015‐2018.

Prevalence of use and the number of poisonings and deaths related to antipsychotics remained relatively stable throughout the study period with the exceptions of quetiapine and olanzapine (Figure 1).

Quetiapine had the highest prevalence of use of all antipsychotics as well as the highest number of poisonings and associated deaths over the study period, followed by olanzapine. We observed a 9.1% relative increase in the annual prevalence of quetiapine use for the period 2015 to 2020 (from 5.6 to 6.10 per 1000 population) and a 4.5% relative decrease in the annual prevalence of olanzapine use (from 3.9 to 3.7 per 1000 population) (Figure 1).

Simultaneously, there were 30.8% and 32.7% relative increases in the number of poison centre calls related to quetiapine (from 1639 to 2144) and olanzapine (from 520 to 690), respectively, but we observed little change in quetiapine‐ and olanzapine‐related deaths for the period 2015 to 2018. In 2018, the highest rates of co‐ingestion in poisoning of other sedating agents were for quetiapine (opioids 7.0% poisonings and 64.0% of poisoning deaths, benzodiazepines 16.8% and 60.9%, pregabalin 3.8% and 16.8%) and olanzapine (opioids 5.1% and 63.5%, benzodiazepines 21.2% and 62.9%, pregabalin 4.9% and 17.6%). The lower rates for other antipsychotics are shown in Table 1.

**TABLE 1:** (A) Number of antipsychotic dispensings with a concurrent dispensing^a^ of an opioid, benzodiazepine or pregabalin in 2020. (B) Number of antipsychotic‐related poisoning calls to the New South Wales Poisons Information Centre in 2020 associated with co‐ingestion of an opioid, pregabalin or benzodiazepine. (C) Number of antipsychotic deaths in the National Coronial Information System in 2018 associated with co‐ingestion of an opioid, pregabalin or benzodiazepine.

(A) Antipsychotic dispensings (2020)	Total	Opioid, n (%)	Benzodiazepine, n (%)	Pregabalin, n (%)
Quetiapine	15 925	4834 (30.4)	4815 (30.2)	1534 (9.6)
Olanzapine	10 005	2220 (22.2)	2797 (28.0)	610 (6.1)
Risperidone	5771	1443 (25.0)	1294 (22.4)	288 (5.0)
Aripiprazole	2685	454 (16.9)	533 (19.9)	133 (5.0)
Clozapine	1530	144 (9.4)	281 (18.4)	39 (2.5)
Other antipsychotics	7441	2884 (38.8)	2032 (27.3)	709 (9.5)

(B) Poisonings (2020)	Total	Opioid, n (%)	Benzodiazepine, n (%)	Pregabalin, n (%)
Quetiapine	2144	150 (7.0)	361 (16.8)	81 (3.8)
Olanzapine	690	35 (5.1)	146 (21.2)	34 (4.9)
Risperidone	358	≤10 (≤3)	16 (4.5)	≤10 (≤3)
Aripiprazole	164	≤10 (≤6)	18 (11.0)	≤10 (≤6)
Clozapine	139	≤10 (≤7)	≤10 (≤7)	≤10 (≤7)
Other antipsychotics	439	11 (2.5)	75 (17.1)	19 (4.3)

(C) Deaths (2018)	Total	Opioid, n (%)	Benzodiazepine, n (%)	Pregabalin, n (%)
Quetiapine	225	144 (64.0)	137 (60.9)	38 (16.9)
Olanzapine	159	101 (63.5)	100 (62.9)	28 (17.6)
Risperidone	22	11 (50.0)	11 (50.0)	≤10 (≤45)
Aripiprazole	29	14 (48.3)	15 (51.7)	≤10 (≤34)
Clozapine	19	≤10 (≤53)	≤10 (≤53)	≤10 (≤53)
Other antipsychotics	68	34 (50.0)	33 (48.5)	12 (17.6)

*Note*: Numbers are expressed as percentages of the total number of dispensings, poisonings or deaths with each antipsychotic. For privacy reasons, cells containing 10 people or fewer have been reported as ≤10.

^a^ Defined as a dispensing in 30 days prior to or following an antipsychotic dispensing.

**FIGURE 2:**
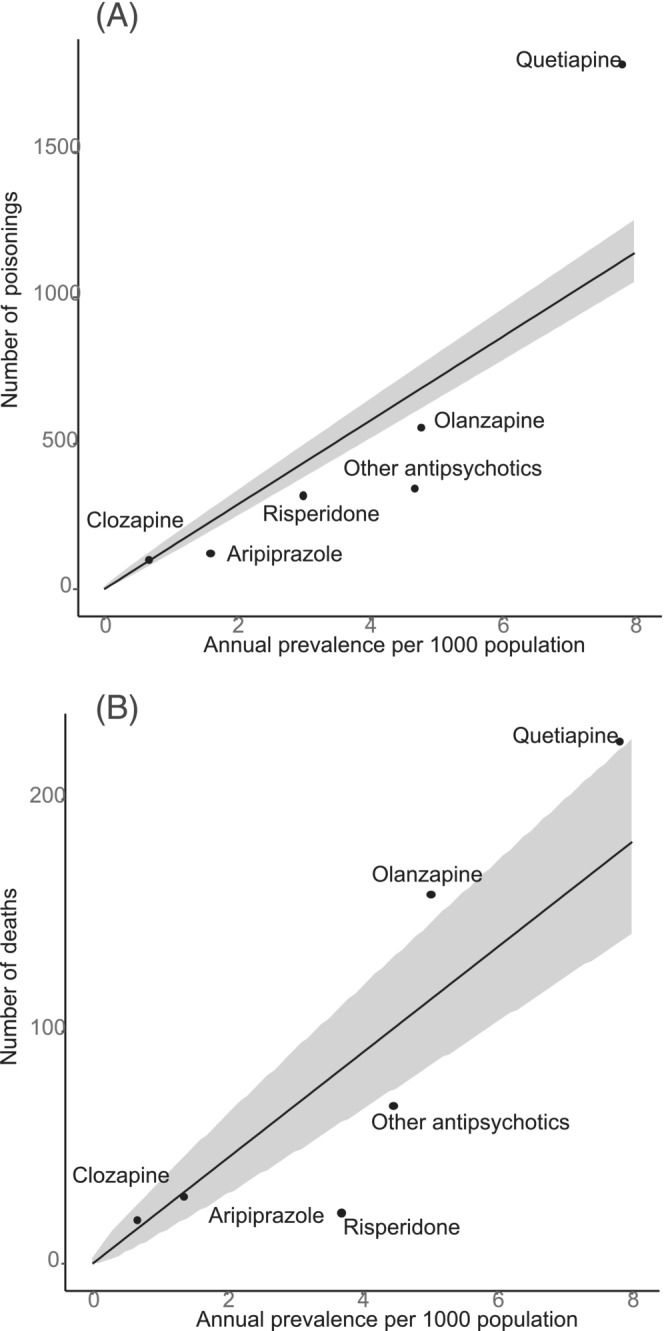
(A) Number of poisonings versus annual prevalence for each antipsychotic in 2020 and (B) number of deaths versus annual prevalence for each antipsychotic in 2018. The solid line represents a Poisson regression of counts (poisonings or deaths) with prevalence as an offset. The grey shaded area represents 95% prediction intervals of the Poisson model.

Relative to prevalence of use, quetiapine was consistently associated with more poisonings and deaths than other antipsychotics (Figure 2). In contrast, all other antipsychotics except clozapine were associated with relatively fewer poisonings and risperidone and “other antipsychotics” were associated with fewer deaths relative to prevalence of use. Most points fell outside the 95% prediction intervals for the Poisson model, suggesting differences between the antipsychotics were not simply random variation, but we did not account for variation by age and sex in these exploratory analyses.

### 3.2 Antipsychotic dispensing patterns

The LCA identified six groups of people dispensed antipsychotics according to their claims‐based antipsychotic indicators and concomitant medicine use, and we further characterized each group by the types of antipsychotics used and the distribution of sociodemographic characteristics (Table 2).

**TABLE 2:** Latent class analysis of antipsychotic dispensing based on indicators relating the type and nature of antipsychotic and sedative dispensing, along with covariates and demographic characteristics.

Group	1: Ongoing high‐dose use with sedatives	2: Ongoing use	3: Ongoing use with analgesics and sedatives	4: Long‐term low‐dose use	5: Sporadic use	6: Sporadic use with analgesics
Total n^a^ (%)	34 484	180 202 (42)	47 930	40 145	85 381	45 429
	(8)	(42)	(11)	(9)	(20)	(10)
Covariate distribution						
Age group, years						
18‐39	30.0%	30.6%	22.5%	26.2%	40.6%	17.4%
40‐64	54.8%	48.1%	50.6%	32.8%	31.6%	25.8%
65+	15.1%	21.3%	26.8%	41.1%	27.8%	56.8%
Female	44.3%	47.7%	57.1%	57.5%	52.3%	56.5%
Concessional beneficiary	90.0%	79.4%	87.2%	78.0%	68.9%	82.9%
Antipsychotic						
Quetiapine	24.1%	30.6%	43.2%	48.2%	47.1%	31.8%
Olanzapine	34.6%	27.9%	24.0%	24.9%	14.9%	8.7%
Risperidone	4.1%	13.3%	9.7%	12.9%	17.3%	16.9%
Clozapine	17.0%	4.6%	1.8%	0.9%	0.2%	0.0%
Aripiprazole	7.0%	9.3%	5.7%	2.4%	4.2%	1.8%
Other	13.3%	14.3%	15.6%	10.7%	16.3%	40.8%
Dispensing indicator distribution						
Mean time between dispensings/year						
Only one dispensing	0.0%	1.6%	0.0%	0.0%	62.0%	57.6%
<20 days	46.7%	3.9%	11.5%	6.3%	3.6%	8.7%
≥20 days	53.3%	94.5%	88.5%	93.7%	34.4%	33.7%
Number antipsychotic prescribers/year						
1 to 3	59.2%	94.7%	90.9%	95.2%	100.0%	100.0%
4+	40.8%	5.3%	9.1%	4.8%	0.0%	0.0%
Number of defined daily doses/year						
<42	0.0%	0.3%	2.1%	57.5%	99.7%	98.4%
43‐330	12.7%	75.3%	71.0%	42.5%	0.3%	1.6%
>330	87.3%	24.4%	26.9%	0.0%	0.0%	0.0%
Only lowest strength/year^b^	0.0%	0.0%	0.2%	50.3%	34.2%	20.9%
Co‐medication/year^c^						
Opioid	23.1%	10.2%	88.1%	33.1%	0.1%	84.6%
Benzodiazepine	43.1%	21.9%	64.0%	35.0%	18.0%	35.1%
Pregabalin	3.6%	1.4%	26.0%	6.7%	1.2%	15.7%

*Note*: Numbers represent the percentage probabilities of membership to a given group. Group names were assigned based on clinical interpretation of indicator and covariate distributions. Total numbers in each class and covariate distributions are based on 2020 data only. Concessional beneficiary: people receiving greater medicine subsidy, aged ≥65 years, on low incomes, unemployed or with disabilities.

^a^ Numbers are the national level pseudo‐counts extrapolated from the Pharmaceutical Benefits Scheme 10% sample estimated from probability of class membership.

^b^ Only included for individual antipsychotics in which the lowest strength tablet yielded a daily dose below the defined therapeutic range according to the Australian Medications Handbook.

^c^ Defined as dispensing within 30 days of the antipsychotic.

“Ongoing high‐dose antipsychotic use with sedatives” (Group 1) accounted for 8%, “ongoing antipsychotic use” (Group 2) 42% and “ongoing antipsychotic use with analgesics and sedatives” (Group 3) 11% of all antipsychotic use (Table 2). Members of Group 1 had the highest antipsychotic volumes dispensed, the shortest time between dispensings, the greatest number of prescribers (four or more prescribers in 40.8%) of any group and the second highest rates of concomitant benzodiazepine use (43.1%) (Table 2). Members of Group 1 tended to be 40‐65 years old, male and were most likely to be concessional beneficiaries. Compared to other groups, they were most likely to receive olanzapine (34.6%) and clozapine (17%). Members of Group 2 were most likely to have 20 days or more between dispensings, relatively unlikely to have multiple prescribers and less likely than Groups 1 and 3 to receive sedatives or analgesics. They were also predominantly aged 40‐64 years, male and about equally likely to receive quetiapine or olanzapine (30.6% and 27.9%, respectively). While aripiprazole was not commonly used in any group, its use was most frequent among people in Group 2. Those in Group 3 sat between Groups 1 and 2 in terms of time between dispensings, volume of use and number of prescribers, but were the most likely of any group to receive opioids (88.1%), benzodiazepines (64.0%) and pregabalin (26%). They were predominantly aged 40‐64 years and female, and most likely to receive quetiapine (43.2%).

“Long‐term low‐dose antipsychotic use” (Group 4) accounted for 9%, “sporadic antipsychotic use” (Group 5) 20% and “sporadic antipsychotic use with analgesics” (Group 6) 10% of all antipsychotic use. People in these groups tended to be female, those in Groups 4 and 6 tended to be 65 years or older and those in Group 4 were 18‐39 years. Members of Group 4 tended to be dispensed the lowest antipsychotic strength only (50.3%) and have 20 days or more between dispensings (93.7%). They were the most likely of any group to receive quetiapine (48.2%). Members of Group 5 were the most likely to receive only one dispensing (62.0%) and were also highly likely to receive quetiapine (47.1%). Compared to other groups, members of Group 6 were also likely to receive only one dispensing (57.6%) and were most likely to receive “other antipsychotics” (most commonly haloperidol) as well as concomitant opioids (84.6%), benzodiazepines (35.1%) and pregabalin (15.7%).

## 4 DISCUSSION

This study represents the largest, most recent Australian study examining population‐based trends in antipsychotic use, poisonings and deaths. It provides a novel perspective on probable therapeutic use based on people's sociodemographic and dispensing characteristics. Quetiapine and olanzapine accounted for the highest prevalence of use, as well as the highest number of associated poisonings and deaths throughout the study period, consistent with previous evidence.^26^, ^27^, ^28^, ^29^ While most antipsychotic use appears to be for psychotic disorders, our data suggest that a substantial proportion of people treated with antipsychotics may not have enduring psychotic symptoms and that quetiapine use is more likely in this population.

Those with ongoing high‐dose antipsychotic use with sedatives and the preponderance of olanzapine and clozapine use (Group 1) is indicative of a population with treatment‐resistant psychosis. Treatment resistance is known to occur in about 30% of people with schizophrenia.^30^ While additional sedative use may be understandable in treatment‐resistant settings, benzodiazepine use has been shown to increase mortality among people with schizophrenia.^31^ The reasons for this group being more likely to have four or more prescribers is unclear and may in part relate to more frequent healthcare presentations. People with the highest rates of benzodiazepine, opioid and pregabalin prescribing (Group 3) may either be experiencing treatment‐resistant psychosis or other difficult‐to‐treat comorbidities such as mood disorders or chronic pain. Chronic pain is more common in people with mood disorders compared to those with schizophrenia and so there may be a preponderance towards bipolar affective disorder and treatment‐resistant depression in this population.^32^, ^33^ Quetiapine and olanzapine were the most used antipsychotics within this group and were also associated with the highest number of poisonings and deaths involving benzodiazepines, opioids and pregabalin.^7^, ^9^ While they are the most prevalent antipsychotics used overall, this does raise concerns about the risk of overdose and death with their use alongside other sedatives.

Off‐label use of quetiapine and to a lesser extent olanzapine and other antipsychotics for indications such as insomnia and anxiety is well documented.^4^ Studies from the United States estimate that between 17% and 42% of second‐generation antipsychotic prescribing is off‐label^4^, ^34^ for conditions where the balance of risk and benefit is unclear or unfavourable. In particular, ongoing dispensing of the lowest strength formulation of quetiapine (Group 4) has been associated with off‐label use for insomnia and anxiety as well as metabolic adverse effects.^6^, ^35^ Sporadic antipsychotic use in a younger cohort (Group 5) may also be for off‐label indications as psychosis‐related indications usually require longer‐term treatment. Sporadic use in an older population with a preponderance of analgesics, sedatives and haloperidol (Group 6) may in part be for behavioural and psychological symptoms of dementia (BPSD). Quetiapine and haloperidol and are commonly used off‐label for this indication and risperidone is registered for less than 12 weeks of use in Australia for BPSD as a last resort.^36^ However, previous research has estimated that antipsychotics increase the risk of death in older adults, with numbers needed to harm of 50 (95% confidence interval [CI] 30‐150), 27 (95% CI 15‐99) and 26 (95% CI 19‐46) for quetiapine, risperidone and haloperidol, respectively.^13^ There are also concerns that inadequate training, staffing and access to nonpharmacological treatments in residential aged care are contributing to antipsychotic prescribing.^37^, ^38^ While rates of sedating analgesic prescribing in this elderly population may reflect comorbidity with pain, such prescribing is associated with increased adverse events such as falls and decline in mobility and cognition.^13^, ^39^

Unlike previous studies of opioids and pregabalin,^17^, ^18^ our LCA did not reveal a distinct group with convincing signs of antipsychotic misuse, even among those with the highest volume dispensing, number of prescribers and concomitant sedative or analgesic use indicators. Instead, higher indicator scores were found in both Groups 1 and 3, raising the question of whether these groups contain people misusing prescribed antipsychotics.^16^

After reports of off‐label use and misuse of quetiapine along with higher rates of overdose and death relative to other antipsychotics, the Therapeutic Goods Administration of Australia increased regulatory control by up‐scheduling quetiapine along with pregabalin in April 2020; the impact of this change has yet to be evaluated. In addition, quetiapine is already included in a number of prescription monitoring programmes across Australia.^40^ However, according to our analyses, indicators to detect potential misuse based on high volumes and overlapping prescriptions alone may lack sensitivity to detect potential misuse and possibly have a high rate of false positives, leading to further stigmatization of already vulnerable groups.

The limitations of this study include a lack of clinical information relating to intended prescribing indication, comorbidities and health service utilization, meaning we were unable to determine with certainty the therapeutic intent of antipsychotic use and the clinical context in which they were prescribed. In addition, private prescriptions are not captured within PBS data and so the overall dispensings of medicines may be underestimated in this study. We were not able to stratify poisonings by age as this was not consistently collected. Long‐acting depot antipsychotics were omitted from dispensing claims analyses and so the groups here do not reflect use of these preparations. Calls to the NSW Poisons Information Centre do not capture all poisonings relating to antipsychotics across NSW, only those in which a call to the Poison Centre was made and only age group rather than age was available. Coronial death data were only presented up to 2018 due to declining case completion rates after this date, limiting contemporary comparisons with PBS and poisoning data.

## 5 CONCLUSIONS

Quetiapine and olanzapine were the most used antipsychotics and most associated with poisonings and deaths, particularly in combination with opioids, benzodiazepines and pregabalin, supporting their inclusion in prescription monitoring systems. However, care should be taken when using algorithms to detect those at risk of misuse and responses to such algorithms should factor in additional contextual clinical information. Suboptimal antipsychotic use for a range of off‐label indications has been well documented previously, but this study has allowed the magnitude of such use to be quantified on a national level. The finding that a substantial proportion of people are likely receiving antipsychotics for nonpsychotic and off‐label conditions underscores the need for improved understanding of the systemic drivers of suboptimal prescribing to facilitate evidence‐based policy action.

## AUTHOR CONTRIBUTIONS

J.B. conceived the analysis, performed analysis of poisons and death data and wrote the manuscript. M.B.G. performed the analysis of PBS data. N.A.B., S.‐A.P., H.Z. and M.B.G. provided feedback on the manuscript and H.Z. had overall oversight of the project.

## CONFLICT OF INTEREST STATEMENT

The authors declare that there is no conflict of interest.

## Supporting information


**SUPPORTING INFORMATION TABLE S1** Antipsychotics included in analyses, along with ATC codes
**SUPPORTING INFORMATION TABLE S2** Antipsychotics with a lowest dose tablet strength that is below the therapeutic dose listed in the Australian Medicines Handbook

## Data Availability

Research data are not shared.
