# Supplementary material for: Patterns of suboptimal antipsychotic use and misuse in Australia: What can routinely collected data tell us?
Source: Br J Clin Pharmacol. 2023 Jul 21;89(11):3411–20. doi: 10.1111/bcp.15821 (PMC10953398; doi:10.1111/bcp.15821)
Supplement: Supplementary file 1 — SUPPORTING INFORMATION TABLE S1 Antipsychotics included in analyses, along with ATC codes SUPPORTING INFORMATION TABLE S2 Antipsychotics with a lowest dose tablet strength that is below the therapeutic dose listed in the Australian Medicines Handbook [file BCP-89-3411-s001.docx]

**SUPPLEMENTARY**

**Latent Class Analysis detailed methods section**

We followed the steps below to complete latent class analyses:

1. Define indicators

We defined cut-points for our indicators that balanced the number of observations in each category, based on indicators calculated for all antipsychotic dispensings over the study period (2015-2020).

2. Fit LCA models

We performed 50 rounds of latent class analysis with different random seeds, applying a data-derived stabilising prior with a strength of one to the rho (ρ) parameters (the item-response probabilities conditional on latent class membership). We grouped the observations by calendar year and applied measurement invariance across these groups. We allowed for a maximum of 25000 iterations of the expectation maximisation estimation procedure to reach convergence.

We only retained models for which at least 20% of rounds converged to the best-fitted model (a measure of fit robustness).

We repeated the LCA fitting for 2 to 6 classes, calculating Akaike’s information criterion (AIC) for each.

3. Compare model metrics and choose the optimal number of classes

We chose the optimal fit (determining the final number of classes) by comparing AICs, while considering if classes had easily discernible differences in indicator and covariate patterns.

4. Determine distribution of other covariates for each class

After performing the LCA, we used the class membership probabilities for each observation to characterise the distribution of other covariates of interest within each class, summing the probability-weighted counts. These covariate distributions assisted in inferring the probable patient types.

5. Characterise classes

Following review by the two clinical pharmacologist authors (JB and NB), we named the six classes using descriptors with clinical face validity, based on the distribution of latent class indicators and covariates. After assigning descriptors we reassessed our choice of optimal fit in step 3.

We performed analysis using SAS version 9.4, PROC LCA and R version 4.0.4 (The Methodology Center Penn State, 2015; R Core Team, 2021).

**Table S1:** Antipsychotics included in analyses, along with ATC codes

| **Antipsychotics Included** | **ATC code** |
| --- | --- |
| Quetiapine | N05AH04 |
| Olanzapine | N05AH03 |
| Risperidone | N05AX08 |
| Aripiprazole | N05AX12 |
| Clozapine | N05AH02 |
| Other antipsychotics |  |
| Amisulpride | N05AL05 |
| Asenapine | N05AH05 |
| Brexpiprazole | N05AX16 |
| Chlorpromazine | N05AA01 |
| Fluphenazine | N05AB02 |
| Haloperidol | N05AD01 |
| Lurasidone | N05AE05 |
| Paliperidone | N05AX13 |
| Periciazine | N05AC01 |
| Trifluoperazine | N05AB06 |
| Ziprasidone | N05AE04 |

**Table S2:** Antipsychotics with a lowest dose tablet strength that is below the therapeutic dose listed in the Australian Medicines Handbook (AMH).

| **Antipsychotic** | **Lowest tablet strength** | **AMH Therapeutic dose** |
| --- | --- | --- |
| Brexpiprazole | Tablet 1 mg | 2-4mg daily |
| Chlorpromazine | Tablet 10 mg | 500-600mg daily |
| Clozapine | Tablet 25 mg | 200-600mg daily |
| Olanzapine | Tablet 2.5 mg | 5-20mg daily |
| Quetiapine | Tablet 25 mg | 400-800mg daily |
